# High throughput automatic muscle image segmentation using parallel framework

**DOI:** 10.1186/s12859-019-2719-3

**Published:** 2019-03-28

**Authors:** Lei Cui, Jun Feng, Zizhao Zhang, Lin Yang

**Affiliations:** 1 Department of Information Science and Technology, Northwest University, Xi’an, China; 2 Department of Computer and Information Science and Engineering, University of Florida, Gainesville, FL USA

**Keywords:** Muscle image segmentation, Cloud computing, Multi-core programming

## Abstract

**Background:**

Fast and accurate automatic segmentation of skeletal muscle cell image is crucial for the diagnosis of muscle related diseases, which extremely reduces the labor-intensive manual annotation. Recently, several methods have been presented for automatic muscle cell segmentation. However, most methods exhibit high model complexity and time cost, and they are not adaptive to large-scale images such as whole-slide scanned specimens.

**Methods:**

In this paper, we propose a novel distributed computing approach, which adopts both data and model parallel, for fast muscle cell segmentation. With a master-worker parallelism manner, the image data in the master is distributed onto multiple workers based on the Spark cloud computing platform. On each worker node, we first detect cell contours using a structured random forest (SRF) contour detector with fast parallel prediction and generate region candidates using a superpixel technique. Next, we propose a novel hierarchical tree based region selection algorithm for cell segmentation based on the conditional random field (CRF) algorithm. We divide the region selection algorithm into multiple sub-problems, which can be further parallelized using multi-core programming.

**Results:**

We test the performance of the proposed method on a large-scale haematoxylin and eosin (H &E) stained skeletal muscle image dataset. Compared with the standalone implementation, the proposed method achieves more than 10 times speed improvement on very large-scale muscle images containing hundreds to thousands of cells. Meanwhile, our proposed method produces high-quality segmentation results compared with several state-of-the-art methods.

**Conclusions:**

This paper presents a parallel muscle image segmentation method with both data and model parallelism on multiple machines. The parallel strategy exhibits high compatibility to our muscle segmentation framework. The proposed method achieves high-throughput effective cell segmentation on large-scale muscle images.

## Background

Skeletal muscle has been extensively recognized as the tissue related to many diseases such as heart failure and chronic obstructive pulmonary disease (COPD) [1, 2]. To accelerate the disease diagnosis at the cellular level and reduce the inter-observer variations, these exist increasing demands for accurate and efficient computer-aided muscle image analysis system [3]. Automatic muscle cell segmentation is usually the first step for further image feature quantification. In recent years, several state-of-the-art algorithms have been reported for cell segmentation on skeletal muscle and various cancer images [4–10]. For example, unsupervised methods, such as the deformable model [4, 10, 11], Liu et al. [4] propose a deformable model-based segmentation algorithm, which uses color gradient for cell boundary seeking. Later a contour detection and region-based selection algorithm, which is able to deal with low quality skeletal muscle images, is presented in [12]. However, due to the high model complexity, these methods are not applicable to large-scale muscle images (e.g. 4000×4000).

Recently, there is an encouraging evidence that applying medical image analysis [13, 14] to high performance computing resources can significantly improve the running time of the algorithms. Meanwhile, analyzing the whole-slide images can provide much richer information, which is helpful to clinical diagnosis [15]. Therefore, there is an urgent need of efficient large-scale image analysis algorithms. High performance computing techniques emerge as one solution to tackle this challenge, and have attracted a great deal of research interests in medical image analysis [14, 16, 17]. In particular, we have successfully applied a cloud computing framework [13, 18, 19] to content-based sub-image retrieval on whole-slide tissue microarray images, and another application is reported in [14] for high throughput landmark based image registration. Although many high performance computing applications in medical image analysis have been presented in recent literatures, there exits very few reports focusing on cell segmentation.

**Fig. 1:**
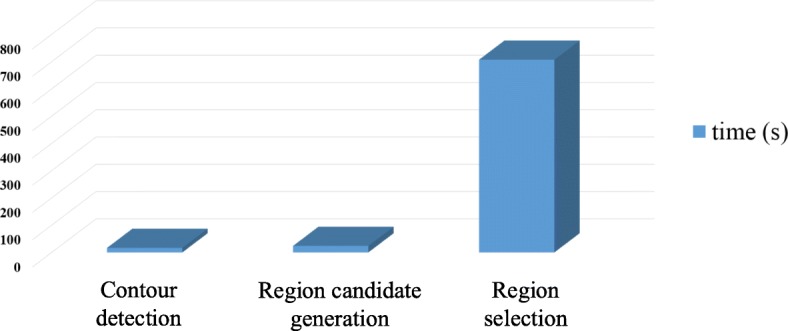
The time profile for each step of the proposed entire segmentation algorithm running on a standalone machine with a 6000×6000 image. The hierarchical tree based region selection step dominates the running time, around 94% of the total time cost

In this paper, we first present an effective muscle cell segmentation framework, mainly consisting of three steps: 1) muscle cell contour detection using structured random forests (SRF); 2) region candidate generation using superpixel techniques, and 3) hierarchical tree based region selection. A similar framework is first presented in our previous conference version [12], and we extend this approach to a distributed computing framework in this paper. Figure 1 shows the time profile of each step of the framework running on a standalone machine. It indicates that the region selection dominates the running time (accounting for around 94%), this paper mostly focuses on accelerating this step with both data and model parallelism.

We propose a parallel approach using cloud computing techniques which is able to handle very large-scale muscle images. A master-worker parallelism manner is exploited to distribute image data onto multiple worker nodes of a cloud cluster. On each worker node in the cluster, we propose a hierarchical tree based region selection leveraging on the conditional random field (CRF) algorithm. Its optimization process is divided into multiple sub-problems, which can be solved using multi-core programming techniques. Our proposed method achieves more than 10 times speed improvement on very large-scale muscle images containing hundreds to thousands of cells. Meanwhile, our proposed method produces superior segmentation results compared with several state-of-the-art muscle image segmentation methods on our H &E skeletal muscle image dataset.

The rest of the paper is constructed as follows: we start by introducing our muscle image segmentation method and analyze its characteristics for parallelism; then we present the parallel approach to accelerate the overall segmentation efficiency; next, the “Experimental results” section evaluates the speed and accuracy of our proposed muscle image segmentation method; the last section concludes this paper.

## Contour detection and region candidate generation

**Fig. 2:**
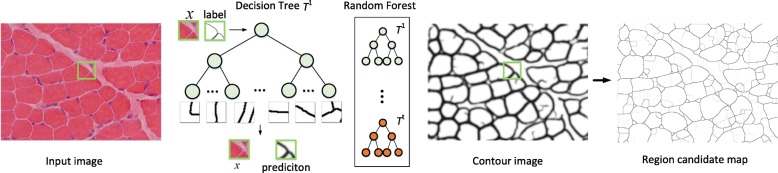
Illustration of the contour detection and region candidate map generation. For each local patch from input test image, our SRF detector outputs a contour prediction patch. The contour image is generated by averaging all pixel-wise predictions. Then the region candidate map is obtained by using OWT-UCM, yielding an over-segmented image

We present the proposed cell segmentation method in this section. Effective contour detection is the first step of most region-based image segmentation methods [20–22]. We start by introducing a structured random forest (SRF) based method for fast and accurate muscle contour detection, SRF is selected because its: 1) fast prediction ability for high-dimensional data, 2) robustness to label noise [23], and 3) good support to arbitrary size of outputs. Next, a superpixel algorithm is used to generate region candidates. Finally, we present a hierarchical tree based method to select the optimal candidate regions based on CRF, Fig. 2 shows the entire process.

### Contour detection

Random forest (RF) classifier is an ensemble learning technique which combines *t* decision trees to form a forest $\mathcal {F} = \left \{T_{j}\right \}_{j=1}^{t}$ [24]. Each tree *T*_*j*_ is trained independently and the final classification is determined by applying a majority voting to all the outputs of trees.

However, conventional RF can not capture the inherent contour structures inside local image patches so that it is difficult to obtain satisfactory contour detection performance [25]. In order to capture rich structures of contours during the SRF training, we propose to deploy SRF [26], a variation of RF, to detect the muscle cell contours. SRF is trained with a set of training data $D = \{(x,y) \in \mathcal {X} \times \mathcal {Y}\}$, where $\mathcal {X} = \mathbb {R}^{(d \cdot d) \times c}$ is the feature space of a *d*×*d* image patch, so that each pixel in the image patch is featured by a *c*-dimensional vector. The structured label $y \in \mathcal {Y} \in \mathbb {Z}^{d \cdot d}$ corresponding to *x* is a patch cropped from the ground truth image, which is a binary image having the value of 1 in contour pixels and 0 otherwise.

To enable the training of SRF with structured labels, in node *i* where training data *D*_*i*_ falls, we adopt a mapping function proposed by [26] to map structured labels into a discrete space for each *x*∈*D*_*i*_, which intrinsically consider the contour structure information. Then a split function *h*(*x*,*θ*)=**1**[*x*(*k*)<*τ*] splits and propagates the data *D*_*i*_⊂*X*×*Y* to the left *L* (when *h*=0) or right *R* (*h*=1) substree of node *i*, which is the same as the node splitting procedure of RF. The *τ* and *k* are determined by maximizing the standard information gain criterion *C*_*i*_ at node *i* [24]:

1 $$  C_{i} = H(D_{i}) - \sum\limits_{o \in \{L,R\}} \frac{|D_{i}^{o}|}{|D_{i}|}H\left(D_{i}^{o}\right),  $$

where *H*(*D*_*i*_) is the Gini impurity measure, $H(D_{i}) = \sum _{l} c_{l}(1-c_{l})$. *c*_*l*_ denotes the proportion of data in *D*_*i*_ with label *l*. After the data in *D*_*i*_ is propagated to the child nodes, the above steps are performed recursively until leaf nodes are reached (i.e., the stopping criteria is satisfied [24]). The most representative structural label *y* (close to mean) in each node is stored as its structured prediction [27].

In practice, following [25], we utilize three color channels computed using the CIE-LAB color space. Two gradient magnitude channels are computed with varying amounts of blur (we use Gaussian blurs with *σ*=0 and *σ*=1.5). Additionally, eight orientation channels in two image scales to represent the features of image patches. Such that in total *c*=13 channels in $\mathcal {X}$ are extracted by using optimized code from [28] available online^1^. To prevent overfitting when training SRF, each tree randomly selects a subset of training samples and features for training. In the testing stage (see Fig. 2), since the prediction of each tree for each pixel is independent, we can parallelize this stage using a multi-thread technique [26].

### Region candidate generation

Based on the contour image detected by our SRF contour detector, region candidates can be generated using superpixel techniques, which is able to group similar pixels in terms of color, edge strength (referring to our detected contour image), and spatial cues.

In this paper we use the well-known oriented watershed transform and ultra-metric contour map (OWT-UCM) [29] algorithm to obtain our region candidate maps for three main reasons: 1) it is very efficient to handle large-scale images; 2) regions in a map are well nested at different thresholds; 3) it guarantees that the boundaries of each region are closed and single-pixel wide. These characteristics can facilitate the parallelism of the subsequent proposed hierarchical tree based region selection algorithm. OWT-UCM takes a contour image as input and outputs an over-segmented region candidate map [30], which is illustrated in Fig. 2. The next step is to select those regions using our proposed hierarchical tree-based region selection algorithm.

### Hierarchical tree-based region selection

Given the over-segmented region candidate map, our region selection algorithm aims to select region candidates as final segmentation by merging or discarding the segments in the region candidate maps.

First we build a hierarchal tree structure using the region candidate map. In our hierarchical tree structure, the leaf nodes represent the initial regions of the region candidate map. The regions are pair-wised merged using a simple hierarchical clustering algorithm to construct the tree. The root node of the tree corresponds to the whole image region. Each node in the tree is a candidate region. The tree structure satisfies the “non-overlapping” criteria, a common way for hierarchical tree based segmentation methods [12, 31, 32].

Suppose there are *N* base candidate regions, the total number of nodes in the tree would be 2*N*−1. We denote *R*={*R*_1_,*R*_2_,…,*R*_2*N*−1_} as the region candidate map consisting of a set of region candidates *R*_*i*_. Our goal is to select nodes in the tree as our final muscle cell segments. We show that this can be achieved by the condition random field (CRF) algorithm [33].

CRF has been widely used in image segmentation. It is a probabilistic graphical model aiming at maximizing a posterior given a defined energy function. In our method, the energy function is defined as

2 $$ E(R) = \sum\limits_{i=1}^{2N{+}1} U_{i}(R_{i}) + \sum\limits_{(i,j) \in \hat{R}} V_{i}\left(R_{i},R_{j}\right),  $$

where $\hat {R}$ is the subset of *R* contains all adjacent regions (i.e., any leaf nodes of a common father node) in leaves of the hierarchical tree. *U*_*i*_(*R*_*i*_) is the unary term for region *R*_*i*_, which is a score to evaluate the probability of *R*_*i*_ covering a complete cell segment. We adopt our previously developed method [12] to evaluate *U*_*i*_ by training a cell scoring classifier, which is able to assign a probability value to determine whether a segment is a good region candidate. In brief, a set of features based on multiple cues are proposed to represent the candidate regions and a standard RF classifier is trained to classify the cell regions. *V*_*i*_(*R*_*i*_,*R*_*j*_) is the pair-wise term to evaluate the dissimilarity between two regions *R*_*i*_ and *R*_*j*_. We define *V*_*i*_(*R*_*i*_,*R*_*j*_) as

3 $$ V_{i}\left(R_{i},R_{j}\right) = \mu e^{-B\left(R_{i},R_{j}\right)} \times L\left(R_{i},R_{j}\right),  $$

where *B*(*R*_*i*_,*R*_*j*_) is the boundary strength and *L*(*R*_*i*_,*R*_*j*_) is the boundary length. *μ* is a constant to trade-off the contribution of the two terms. These two terms can be calculated based on the single-pixel wide and closed region candidate maps generated by OWT-UCM [29].

The inference procedure is to minimize the energy function *E* so as to assign a label (1 means this region is a complete cell segment and 0, otherwise) to each region in the node and, at the same time, satisfy the “non-overlapping” criteria, i.e., any substree can only has one label. We deploy the pylon model, a hierarchical CRF model, to minimize *E* [34]. However, the tree will become very big as the number of initial segments inside increases. In the next section, we propose a strategy to divide the inference procedure into several sub-problems which can be parallelized using multi-core programming.

## Parallel muscle image segmentation

**Fig. 3:**
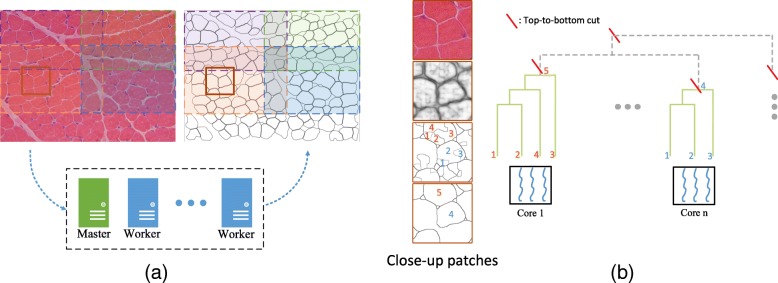
**a**: The partially overlapped tiles (left muscle image) are distributed to workers as tasks. The returned segmentation results are combine to generate the right image. **b**: Close-up patches of the test image in (**a**) is shown. From top to bottom, the four close-up patches are the original image, contour image, initial region candidate map, and the candidate map thresholded by a high value, respectively. The initial candidate map is built into a tree structure. Each region in the high-thresholded candidate map is a small tree using region-wise distance computed using the contour image. The hierarchical tree based inference algorithm is parallelized using multi-core techniques

In this section, we present the proposed data distributed and model parallelized approach for muscle cell segmentation. We first introduce the data distribution procedure, which assigns the data (i.e., non-overlapped image tiles) to multiple workers using a master-worker parallelism manner. Then we introduce the method to parallelize the proposed hierarchical tree based region selection method using multi-core programming. Figure 3 illustrates the two steps.

### Data distribution using spark

Due to the extremely high resolution of muscle images, the running time cost on a standalone machine is computational expensive. Since the segmentation of different image regions is independent with each other, we propose to divide the image into multiple partially-overlapped tiles and distribute them onto multiple worker nodes for concurrent processing.

To this end, we implement this parallel strategy in a master-worker manner with the Spark cloud computing platform [35]. In comparison with other distributed computing frameworks, Spark has the following advantages: 1) it has a flexible cluster management mechanism such that a parallel system can be easily built and run on local clusters; 2) it uses an Resilient Distributed Datasets (RDDs) technique [36] to perform in-memory computations, which is suitable for applications requires large storage space; 3) it exhibits strong compatibility, supporting multiple standard programming languages.

Our parallel muscle image segmentation algorithm consists of three steps: 1) data distribution: the test image *I* is divided into *w* tiles, $\mathbb {I}_{1}, \ldots,\mathbb {I}_{w} $, and the master dynamically maps $\mathbb {I}_{w}$ to all worker nodes using a user-defined map function; 2) segmentation: on each worker node, the proposed cell segmentation algorithm will be executed on multi-cores to perform contour detection, region candidate generation, and region selection; 3) data collection: the segmentation results returned by each worker node are collected to form the final segmentation. To avoid the loss of cell segments crossing the stitching positions of different tiles, we simply pad the tiles to make neighborhood tiles partially overlapped (the padding size is empirically set to 300×300). In order to reduce the overhead of data transfer between master-worker and alleviate extra cost of combing results returned from workers, we only require workers to return masked binary images which will be concatenated as the final segmentation results as shown in Fig. 3a.

With above data level parallelism, we can speed up the segmentation algorithm with no more than *K* times (because of data communication overhead) with *K* worker nodes in the cluster. To further speed up our segmentation algorithm, we parallelize the proposed hierarchical tree based region selection algorithm.

### Hierarchical inference in parallel

The proposed hierarchical tree based inference method is mainly composed of: 1) building a tree structure using the region candidate map, 2) extracting feature representation for each *R*_*i*_ in the tree node, 3) computing *U*_*i*_(*R*_*i*_) for each *R*_*i*_, and 4) minimizing the energy function *E*. Based on our experiments, we observe that steps 2 and 3 dominate the time cost when number of nodes in the tree grow to a large size. This is usually owing to two reasons. First, there are a large number of cells in an muscle image. Second, the low muscle image quality causes contour image having many false positive detections, which make the generated region candidate map contain numerous initial over-segments. However, we can still use the intensity of the contour image to evaluate the probability of real cell contours. We cut the tree from top-to-bottom by the region-wise distance computed from the detected contour image. We regard two adjacent regions whose common contour intensity above a certain threshold as two separate cells, and thus this two regions are not necessary to be clustered to a single substree, so as their ancestor nodes. Figure 3b illustrates the idea. Therefore, the tree is separated into several substrees and the energy minimization process (step 4) between substrees is independent. We parallelize the inference algorithm using a multi-core programming technique on all worker nodes.

## Experimental results

In this section, we demonstrate the efficiency of our proposed parallel approach compared with the standalone mode for large-scale muscle image segmentation. We also evaluate the segmentation accuracy compared with other methods on a H &E stained skeletal muscle image dataset, which are captured by the whole-slide digital scanner from the cooperative institution Muscle Miner and the segmentation ground truth is annotated by several experts.

### Data preparation

The images are cropped from a set of whole-slide scanned skeletal muscle images. We evaluate the efficiency of the proposed method using a set of large-scale images (larger than 4500×3500). In addition, we measure the segmentation accuracy with a dataset contains 100 training images and 69 test images. The size of the images is varying from the scale of 600×600 to 2000×2000. The segmentation ground truth is annotated by several experts. Note that we use this dataset for the segmentation accuracy evaluation as the image size of this dataset is adaptable to the competing muscle image segmentation methods.

### Efficiency evaluation

To evaluate the efficiency, we build a small cluster using 8 Linux machines, each with 6 cores (Intel i7@3.60GHz × 6) and 32 GB RAM. Each core is treated as a independent computing unit (worker node). In total we construct a cloud cluster with 48 nodes and 256 GB RAM.

The parallelism of the proposed method has two levels: data level parallelism using cloud computing and model level parallelism using multi-cores. Based on our observation, there is a trade-off between the tile size and the number of tiles (each tile is a task distributed to a worker node in the cluster). Given a test image, the more tiles we have, then the smaller tile size we obtain. If the tile size is too small, the computation duty of a worker node is too slight to maximize the performance of the multi-core parallel hierarchical tree region selection algorithm. Meanwhile, a large number of tiles would bring too much data communication cost. On the other hand, our model level parallelism may have resource (cores of each machine) conflicts with data level parallelism. Practically we use only 2 cores of each machine as worker nodes in the cluster, and thus in total we use a maximum number of 16 worker nodes.

**Fig. 4:**
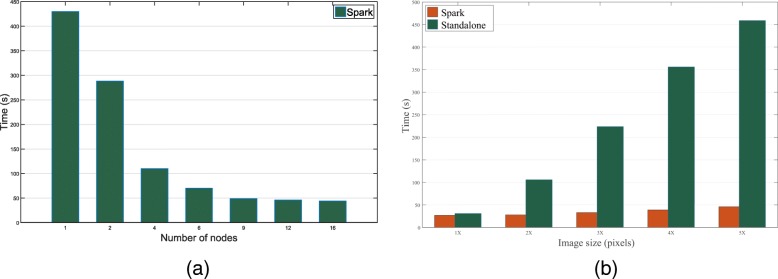
**a**: The running time cost using different number of nodes on Spark. **b**: The comparison of time cost between the proposed parallel method and the standalone version. The x-axis is the image size (1x =1000∗800)

In Fig. 4a, we visualize the time cost using different number of worker nodes in the cluster with a 4600×3800 test image. As we can see, as the number of nodes increases, the time cost drops dramatically. We can achieve a significant speed improvement when the number of node increasing from 1 to 8, but the time decreasing is not obvious from 9 to 12. This is attributed to the trade-off between the size and the number of image tiles, and the data communication overhead. The time cost for data communication will gradually increase as the tile size decreases. In Fig. 4b, we compare the time cost between the Spark based parallel mode and the standalone mode. We can obtain more than 10 times speedup with 5 × (5000×4000) image size.

### Segmentation performance

To evaluate segmentation performance, we report precision, recall and F_1_-score, which is defined as

4 $$ \begin{aligned} & Precision= \frac{|S \cap G|}{|S|}, \qquad Recall= \frac{|S \cap G|}{|G|}, \\ & F_{1}{-}score= 2 \cdot \frac{ Precision \cdot Recall}{Precision+Recall}, \end{aligned}  $$

where *S* is the segmented cell region and *G* is the corresponding groundtruth cell region. |·| means the area of the region. Since the evaluation is cell-wised, for each test image, precision and recall is computed by averaging all cell evaluation results.

**Fig. 5:**
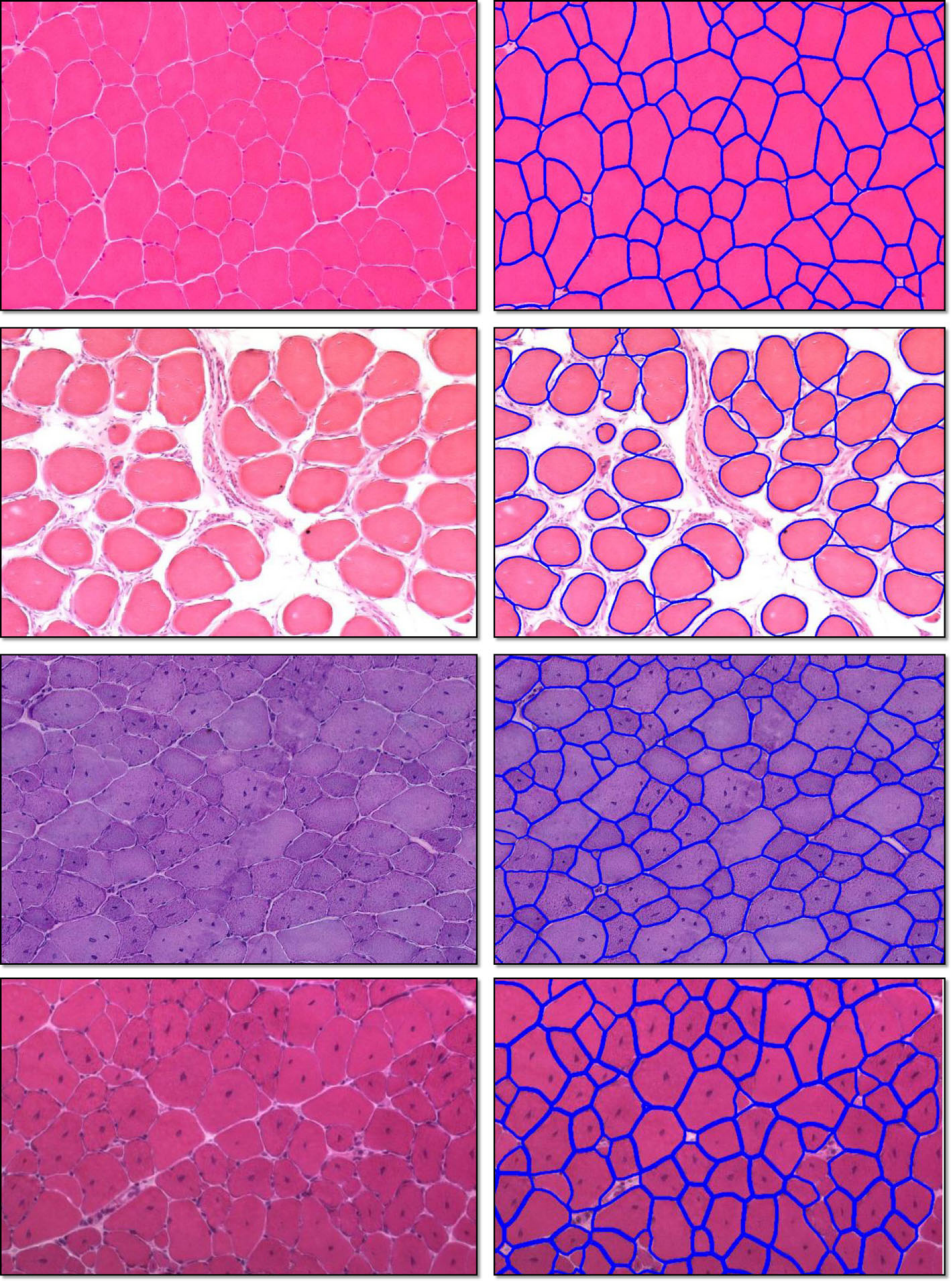
Segmentation results on four sample H&E stained skeletal muscle image patches. The left column is the original images and the right column is the corresponding overlaid segmentation results. The blue lines are the contours of segmented cells overlaid on the original images for better visualization

**Fig. 6:**
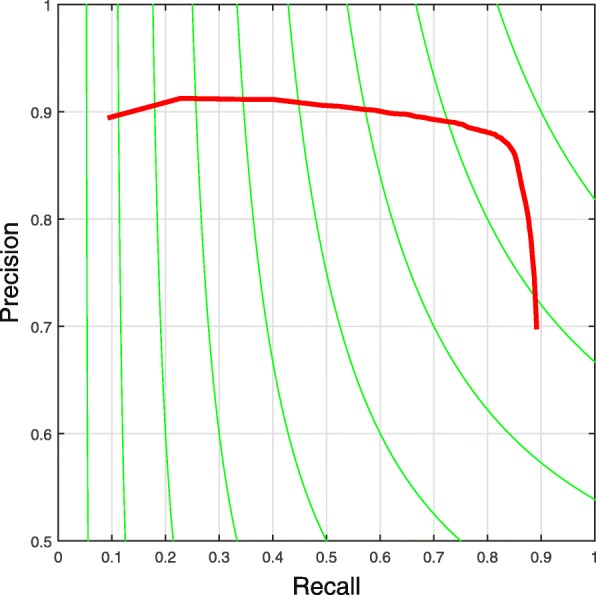
Precision-recall curve on our muscle image dataset, which is drawn by varying the score threshold of the selected candidate regions

Figure 5 shows some the segmentation results, where the test images exhibit significant variations on cell sizes, shapes and appearances. It is clear that the proposed algorithm can accurately segment out most of the individual cell, which demonstrates the robustness of our proposed method. Figure 6 shows the precision-call curve of our method. Our proposed method can preserve high precisions at recalls in a large range, which means that our method is capable to preserve and segment most of the cells in muscle images.

We compare the proposed parallel muscle image segmentation algorithm with two state-of-the-art image segmentation algorithms: 1) gPb [29], which is an edge-based image segmentation algorithm and has been widely used in the image segmentation field. The major drawback is its low efficiency, which takes about 300s for a 1000×100 test image; 2) Isoperimetric graph partition (ISO) [37], which produces high quality segmentations as a spectral method with improved speed and stability. In Table 1, the proposed method outperforms the comparative segmentation approaches. Although gPb performs a high precision, it exhibits very low recall. Compared with these algorithms, our algorithm achieves largely improved recall while exhibits significantly improved running time cost.

**Table 1:** The comparison results of state-of-the-art image segmentation algorithms

Method	F_1_-score (%)		Precision (%)		Recall (%)	
	mean	std	mean	std	mean	std
ISO [37]	80.50	0.0993	89.88	0.0589	74.29	0.1369
gPb [29]	79.04	0.0780	**91.23**	0.0515	70.11	0.0962
Proposed method	**84.61**	0.0134	85.99	0.0035	**85.11**	0.0181

Our results reported here are computed by setting the score threshold of the selected candidate regions to 0.26 (see Fig. 6). Our proposed method shows significantly higher recall than others. These entries in boldface are means the best results

## Conclusion

In this paper, we propose a parallel approach for fast and accurate H &E stained skeletal muscle image segmentation using cloud computing and multi-core programming, which can provide a high throughput solution for computer-aided muscle image analysis with significantly reducing the labor efforts. Specifically, we present a novel muscle image segmentation framework and demonstrate its accessibility to be parallelized. Then a data parallel approach is proposed to accelerate the proposed segmentation method in a master-worker parallelism manner based on the Spark cloud computing platform. To further maximize the computational efficiency on each worker node, we propose to a new strategy to parallelize our proposed hierarchical tree inference algorithm for region selection using multi-core techniques. Experimental results indicate a more than 10 times speed improvement compared with the standalone mode of the proposed segmentation method. Moreover, the comparison results with several competing methods demonstrate the superior performance of the proposed method on our H &E skeletal muscle image dataset.

## Abbreviations

COPD: Chronic obstructive pulmonary disease
CRF: Conditional random field
H&E: Haematoxylin and eosin
ISO: Isoperimetric graph partition
OWT-UCM: Oriented watershed transform and ultra-metric contour map
RDDs: Resilient distributed datasets
RF: Random forest
SRF: Structured random forest

## Footnotes

1
https://github.com/pdollar/toolbox/tree/master/channels
